# Comparison of Feto-Maternal Outcome in Low-Lying Placenta and Placenta Previa in Scarred Versus Unscarred Uterus: A Prospective Observational Study

**DOI:** 10.7759/cureus.108402

**Published:** 2026-05-06

**Authors:** Vaibhav Kanti, Smriti Sagar, Pragati Divedi, Banashree Nath, Nupur Mittal, Snehlata Meena

**Affiliations:** 1 Obstetrics and Gynaecology, Uttar Pradesh University of Medical Sciences, Saifai, IND; 2 Obstetrics and Gynaecology, All India Institute of Medical Sciences, Raebareli, IND

**Keywords:** maternal morbidity, obstetric surgical procedures, placenta previa, postpartum hemorrhage, pregnancy complications, repeat caesarean sections

## Abstract

Introduction: A scarred uterus has a high incidence of defective implantation of the placenta. This being a major cause of third-trimester haemorrhage, we undertook this study to compare the feto-maternal outcome in women with low-lying placenta and placenta previa having scarred and unscarred uterus.

Methods: Women with low-lying placenta and placenta previa at or after 28 weeks of gestation were recruited for the study. A pretested proforma meeting the objectives of the study was prepared, and a detailed history was taken. Maternal and foetal complications were noted and compared between the groups having scarred and unscarred uterus.

Results: The incidence of low-lying placenta and placenta previa was significantly lower in women with unscarred uterus (0.91%) compared to scarred ones (3.20%) (p<0.05). The overall blood loss (p≤0.05) and maternal complication rates, viz. placenta accreta syndrome (p=0.014), postpartum haemorrhage (p=0.002), surgical interventions (p=0.09) to manage these complications, and intensive care unit admission (p=0.009), were high in women with scarred uterus. A woman with a scarred uterus had an odds of 4.11 (95% CI: 1.30-13.56) to undergo massive blood transfusion (>5 units of blood) during delivery. There was a significant inverse linear correlation of blood loss during caesarean section and gestational age at delivery.

Conclusion: Scarred uterus with placenta previa can independently increase the risk of placenta accreta spectrum, postpartum haemorrhage, blood transfusion, and admission to intensive care units. These women have higher odds of undergoing lifesaving interventions. Such interventions at a lower gestational age of delivery are associated with significant postpartum haemorrhage.

## Introduction

Placenta previa is a major obstetric complication characterised by partial or complete implantation of the placenta in the lower uterine segment, covering or approaching the internal cervical os. It remains one of the leading causes of antepartum haemorrhage and is associated with significant maternal and neonatal morbidity and mortality. Globally, placenta previa complicates approximately 0.3-0.5% of pregnancies [1], though its prevalence is rising in parallel with the increasing rates of caesarean sections and other uterine surgeries. Evidence suggests that women with a previous caesarean section have a substantially higher risk, up to 37% in some cohorts, of developing placenta previa in subsequent pregnancies [2].

In low- and middle-income countries, the burden is even more profound. Major obstetric haemorrhage linked to placenta previa accounts for nearly 30% of maternal deaths in parts of Asia [3], emphasising the need for early identification and risk stratification. Alongside maternal risks such as massive haemorrhage, hysterectomy, transfusion, and intensive care unit (ICU) admission, placenta previa is also strongly associated with adverse perinatal outcomes, including preterm birth, low birth weight, and increased neonatal intensive care unit (NICU) admissions [4].

Uterine scarring, defined as histological alteration of the myometrial-endometrial interface following surgical or mechanical trauma, plays a central role in the etiopathogenesis of abnormal placental implantation. Theories explaining defective implantation in scarred uteri include impaired decidualisation, poor vascularisation around the scar niche, and abnormal trophoblastic invasion resulting from disrupted endometrial architecture [5]. Prior uterine procedures such as caesarean section, myomectomy, hysteroscopic adhesiolysis, curettage, and cornual resection for ectopic pregnancy are well-recognised contributors to the formation of uterine scars [5]. As the number of these procedures has increased globally, the incidence of placenta previa and related disorders, particularly placenta accreta spectrum, has shown a parallel rise.

Uterine scarring is well known to be associated with abnormal placentation, and prior studies have evaluated outcomes in placenta previa and low-lying placenta. However, existing evidence is heterogeneous, with limited direct comparisons of feto-maternal outcomes between scarred and unscarred uteri using uniform criteria, particularly in resource-limited settings. In addition, most studies focus on placenta accreta spectrum or caesarean-related morbidity, with less emphasis on broader maternal and neonatal outcomes. Therefore, this study aims to compare feto-maternal outcomes in women with placenta previa and low-lying placenta based on the presence or absence of uterine scarring in a resource-constrained setting.

## Materials and methods

This prospective observational study was conducted in the Department of Obstetrics and Gynaecology of Uttar Pradesh University of Medical Sciences, a tertiary care centre in Saifai, India, over a period of 18 months, from January 2021 to June 2022, after obtaining approval from the institute's Ethical Committee (approval number: 211/2020-21). Written informed consent was obtained from all participants prior to enrolment. The study was reported in accordance with the Strengthening the Reporting of Observational Studies in Epidemiology (STROBE) guidelines.

Study population and recruitment

All pregnant women admitted with a diagnosis of placenta previa at or beyond 28 weeks of gestation were eligible for inclusion, irrespective of parity, type of placenta previa, booking status, or foetal status. Participants were recruited through both emergency and elective pathways.

The study included women presenting to the emergency department with antepartum bleeding who were subsequently diagnosed with placenta previa, as well as those diagnosed during the antenatal period and admitted electively for planned caesarean section. It also included booked antenatal patients who presented with bleeding episodes during pregnancy and were later confirmed to have placenta previa.

Exclusion criteria

Women with antepartum haemorrhage due to causes other than placenta previa, such as abruptio placentae, marginal placental separation, or rare local causes including cervical polyp, varicosities, or genital tract trauma, were excluded. Patients with placenta previa associated with significant co-morbidities such as pre-eclampsia, hyperglycaemia in pregnancy, or severe foetal growth restriction were also excluded.

Data collection

A pretested proforma designed in accordance with the objectives of the study was used for data collection. Detailed history, including maternal age, parity, and number of bleeding episodes during the index pregnancy, was recorded. A comprehensive obstetric and surgical history was obtained, including previous caesarean sections, myomectomy, hysteroscopic procedures for intrauterine adhesions, uterine curettage, and cornual resection for ectopic pregnancy.

Placental localisation

Placental location was assessed antenatally using transvaginal ultrasonography performed by trained physicians, following the diagnostic criteria recommended by the American Institute of Ultrasound in Medicine. A placental edge located more than 20 mm from the internal cervical os was considered normal, a placental edge located less than 20 mm from the internal cervical os was classified as a low-lying placenta, and a placenta completely covering the internal os was diagnosed as placenta previa [6].

Management and blood loss estimation

All patients were managed according to standard obstetric protocols. Intraoperative blood loss during caesarean section was estimated using a combination of methods, such as the following: First is the gravimetric method. Blood-soaked surgical items (gauze, mops, and drapes) were weighed, and the dry weight was subtracted to calculate blood loss, assuming 1 g of weight to be equivalent to 1 mL of blood. Second is the suction bottle measurement. Blood collected in calibrated suction containers was measured after subtracting the volume of irrigation fluids and estimated amniotic fluid. The volume of amniotic fluid was independently estimated by the operating surgeon, assistant surgeon, and assisting nurse, and the mean value was considered for final calculation. Third is the clot measurement. Blood clots were weighed and converted to volume using the assumption that 1 g equals 1 mL of blood. The final blood loss was calculated by summing the values obtained from all methods.

Data were collected using a structured study proforma and entered into a Microsoft Excel spreadsheet (Microsoft Corporation, Redmond, Washington, United States). Statistical analysis was performed using IBM SPSS Statistics for Windows, Version 26.0 (IBM Corp., Armonk, New York, United States), along with appropriate standard statistical methods. Categorical variables were expressed as frequencies and percentages, while continuous variables were presented as mean±standard deviation or median, as appropriate. Data normality was assessed using the Kolmogorov-Smirnov test. Quantitative variables were compared between two groups using the independent t-test or the Mann-Whitney U test, as appropriate. Qualitative variables were analysed using the chi-squared test or Fisher's exact test. Associations between variables were evaluated using appropriate correlation analysis. A p-value of <0.05 was considered statistically significant.

## Results

**Figure 1:**
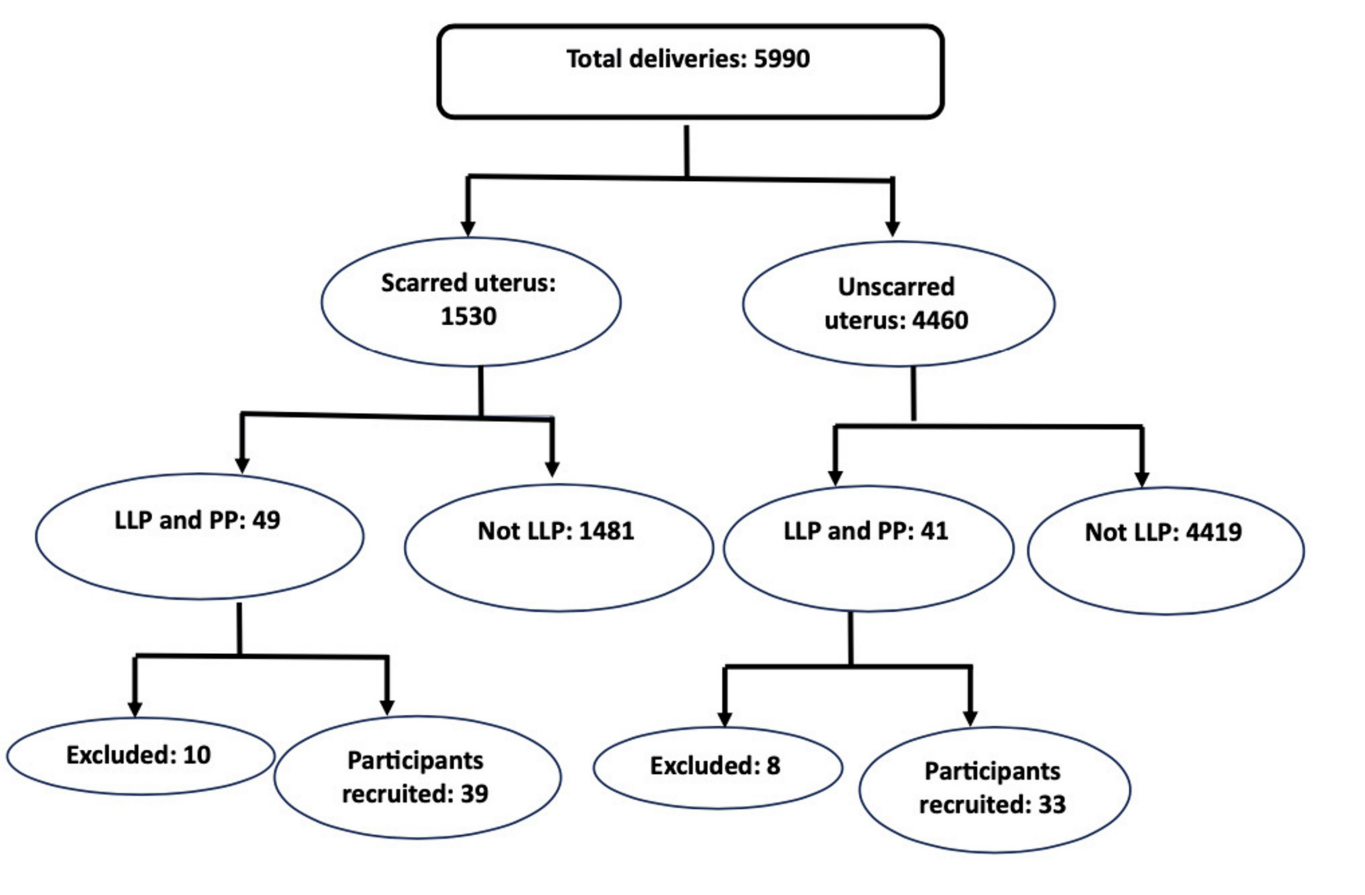
Flowchart showing the selection of cases of placenta previa in scarred and unscarred uterus LLP: low-lying placenta; PP: placenta previa

A total of 5990 patients delivered during the study period of 18 months. The participant enrolment flowchart is depicted in Figure 1.

Out of 5990 pregnant patients, 25.54% had scarred uterus, and the rest (74.46%) had no history of any surgical intervention, either minor or major, ever undertaken in their reproductive organs. Ninety patients had a low-lying placenta; hence, the incidence was 1.5% in our study. The incidence remains significantly low in unscarred uterus (0.91%) compared to scarred ones (3.20%) (p<0.05). After excluding 18 patients not fulfilling the inclusion criterion, a total of 72 patients with low-lying placenta and placenta previa (39: scarred; 33: unscarred) were recruited for the study. The demographic and obstetric profiles of the recruited patients are presented in Table 1.

**Table 1:** Demographic and obstetric characteristics of the patients with placenta previa ^a ^independent t-test; ^b^ chi-squared test; ^c^ Fisher's exact test; ^d^ modified Kuppuswamy socioeconomic scale (here, the upper middle and lower middle are merged into middle socioeconomic status, and the upper lower and lower are merged into lower socioeconomic status) BMI: body mass index; IUD: intrauterine device; ICU: intensive care unit; PAS: placenta accreta syndrome; APH: antepartum haemorrhage; PPH: postpartum haemorrhage; AKI: acute kidney injury; DIC: disseminated intravascular coagulation

Parameters	Scarred (N=39), n (%)	Unscarred (N=33), n (%)	Value of the test statistic	P-value, OR (95% CI)
Demographic variables				
Age (years) (mean±SD)	27.18±4.90	31±6.34	t=-2.819; df=70	0.005^a^
Socioeconomic status^d^				
Upper	3 (7.7)	2 (6)	Fisher's exact test: 10.655; df=2	0.005^c^
Middle	28 (71.8)	12 (36.4)		
Lower	8 (20.5)	19 (57.6)		
BMI (kg/m^2^) (mean±SD)	27.19±3.32	25.50±2.58	t=2.37; df=70	0.021^a^
Gestational age (days) (mean±SD)	254±15.77	250±17.51	t=1.037; df=70	0.303^a^
Duration of marriage (years) (mean±SD)	5.36±2.72	4.97±3.69	t=0.513; df=70	0.609^a^
Obstetric variables				
Gravidity (mean±SD)	3.03±1.08	2.15±1.43	t=2.93; df=70	0.005^a^
Foetal variables				
Foetal weight (kg) (mean±SD)	2.36±0.28	2.37±0.62	t=-0.086; df=70	0.932^a^
Apgar score				
First minute (mean±SD)	7.33±1.96	6.15±2.95	t=2.65; df=70	0.000^a^
Fifth minute (mean±SD)	9.45±1.72	8.42±0.92	t=2.84; df=70	0.007^a^
Foetal outcome				
Survived	34 (87.1)	26 (78.8)	Fisher's exact test: 2.40; df=2	0.301^c^
IUD	4 (10.3)	7 (21.2)		
Stillborn	1 (2.6)	0 (0)		
Maternal complication				
Mean Hb on admission (gm/dL) (mean±SD)	7.71±1.31	7.56±1.69	t=0.414; df=70	0.674^a^
Type of caesarean section				
Emergency	15 (38.5)	18 (54.5)	Chi-square: 1.86; df=1	0.172^b^
Planned	24 (61.5)	15 (45.5)		
Blood loss during caesarean section (mean±SD)	1484±436	946±376	t=5.54; df=70	<0.05^a^
Blood loss				
<1500 ml	22 (56.4)	26 (78.8)	Chi-square: 4.02; df=1	0.045^b^, 2.87 (1.00-8.19)
>1500 ml	17 (43.6)	7 (21.2)		
Blood transfusion	37 (94.9)	27 (81.8)	Chi-square: 3.08; df=1	0.079^b^, 4.11 (0.77-16.99)
Massive blood transfusion (>5 units)	7 (17.9)	2 (6.06)	Fisher's exact test: 2.31; df=1	0.129^c^, 3.39 (0.65-17.60)
Maternal ICU stay	17 (43.6)	5 (15.2)	Chi-square: 6.81; df=1	0.009^b^, 4.32 (1.30-13.56)
PAS	9 (23.1)	1 (3)	Fisher's exact test: 6.00; df=1	0.014^c^, 9.60 (1.14-20.39)
APH	30 (76.9)	26 (78.8)	Chi-square: 0.036; df=1	0.850^b^
PPH	26 (66.7)	10 (30.3)	Chi-square: 9.45; df=1	0.002^b^, 4.60 (1.60-12.46)
AKI	3 (7.7)	0 (0)	Fisher's exact test: 2.64; df=1	0.104^c^
Hysterectomy	8 (20.5)	3 (9.1)	Fisher's exact test: 1.80; df=1	0.180^c^
DIC	4 (10.3)	1 (3)	Fisher's exact test: 1.44; df=1	0.22^c^
Maternal death	6 (15.4)	2 (6.1)	Fisher's exact test: 1.57; df=1	0.210^c^

There was a significant difference between the two groups of patients in terms of age, socioeconomic status, body mass index (BMI), gravidity, and maternal complication rates. Among the 39 with scarred uterus, 32 women had a history of previous caesarean delivery, five had undergone myomectomy, and two had either twice or more endometrial curettage in the past.

The overall blood loss and need for blood transfusion were significantly high in the scarred group. Maternal complication rates were also higher in women with a scarred uterus. These included increased risks of placenta accreta spectrum, postpartum haemorrhage, and the need for ICU admission, which consequently led to a greater requirement for surgical interventions to manage these complications (Table 2).

**Table 2:** Surgical interventions to control postpartum haemorrhage in women with scarred and unscarred uterus *Fisher's exact test

Surgical procedure to control postpartum haemorrhage	Scarred (N=39), n (%)	Unscarred (N=33), n (%)	P-value	OR (95% CI)
Caesarean section with internal iliac artery ligation	11 (28.2)	4 (12.1)	0.09*	2.84 (0.81-10.0)
B-Lynch suture	7 (17.9)	3 (9.1)	0.279*	2.18 (0.51-9.24)
Hayman suture	6 (15.4)	0 (0)	0.019*	1.18 (1.03-1.35)
Hysterectomy	11 (28.2)	2 (6.1)	0.015*	6.08 (1.24-29.88)

**Figure 2:**
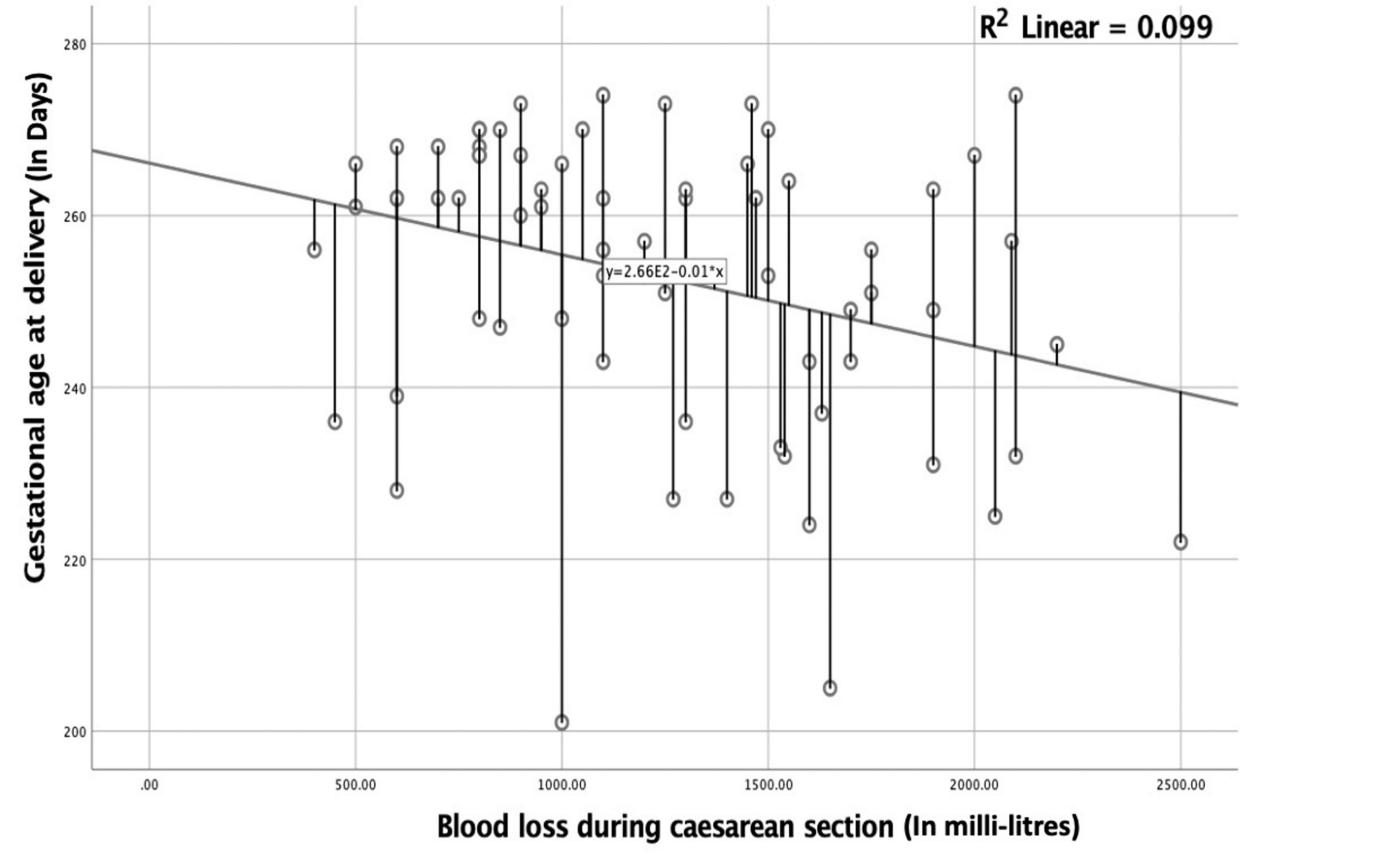
Linear correlation of blood loss during caesarean section and gestational age at delivery of patients with placenta previa Spearman correlation: -0.318; p=0.006

A woman with a scarred uterus had an odds of 4.11 (95% CI: 1.30-13.56) to undergo massive blood transfusion (>5 units of blood) during delivery. There was a significant inverse linear correlation of blood loss during caesarean section and gestational age at delivery (Figure 2).

**Figure 3:**
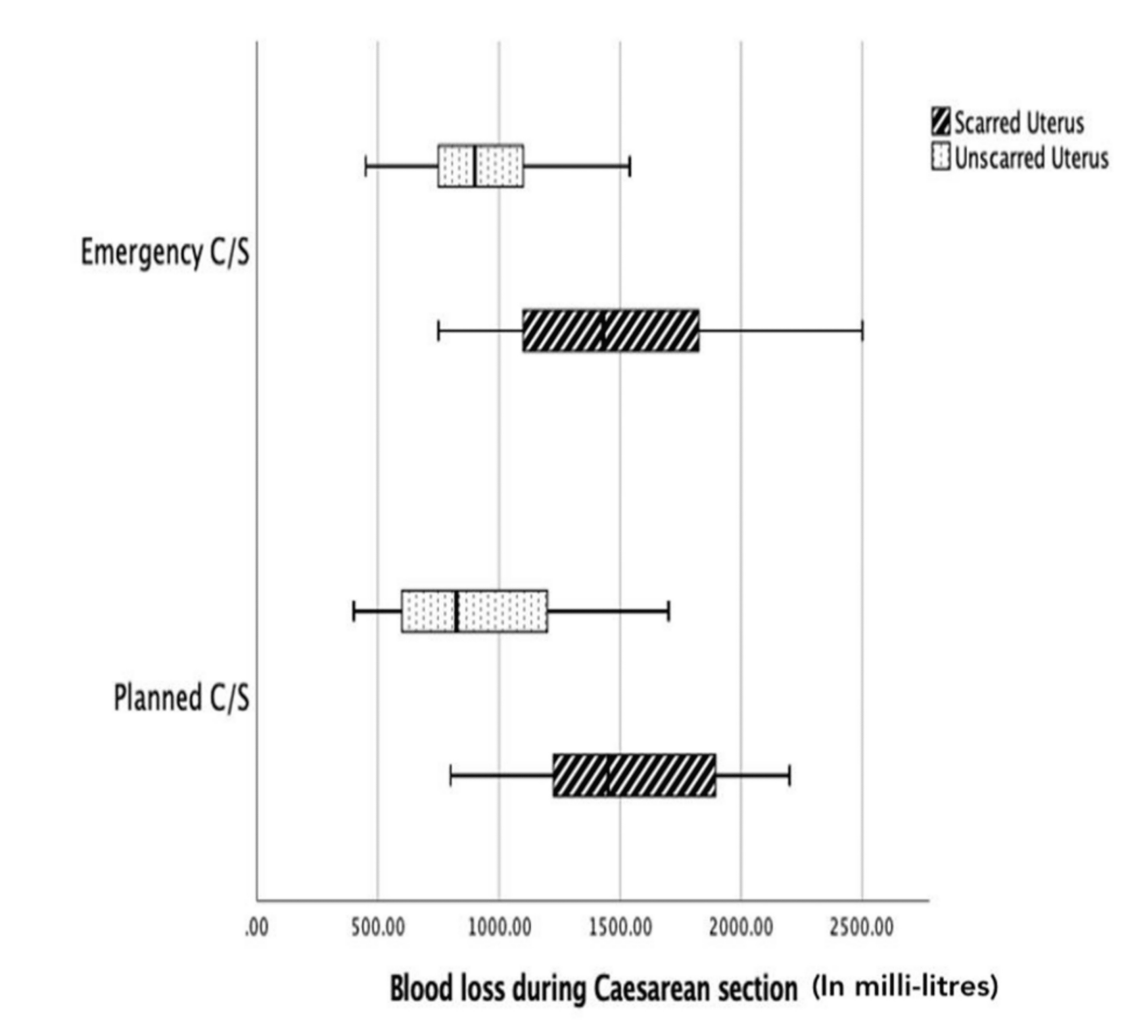
Clustered boxplot of blood loss during caesarean section during emergency and planned procedures in women with scarred and unscarred uterus C/S: caesarean section

The blood loss during emergency caesarean section was high when compared to planned procedures in women with unscarred uterus, but it almost remained the same for scarred uterus (Figure 3).

The foetal complications overall were poor in both groups, with a total of 15.3% of intrauterine foetal deaths (5.6% in scarred and 9.72% in unscarred women). Average Apgar scores at the first and fifth minutes were significantly different in both groups. However, foetal weight was comparable.

## Discussion

A scarred uterus has an increased risk of abnormal placental positioning [2]. Scarred areas in the uterus tend to have poor blood supply, low oxygen levels, and inflammation. These conditions interfere with re-epithelialisation and decidualisation, resulting in the abnormal attachment of placental villi and excessive invasion by trophoblasts [7].

Our study reveals that scarring of the uterus with placenta previa is an independent risk factor for the occurrence of placenta accreta spectrum (OR=9.60; 95% CI: 1.14-20.39), postpartum haemorrhage (OR=4.60; 95% CI: 1.60-12.46), blood transfusion (OR=4.11; 95% CI: 0.77-16.99), and ICU admission (OR=4.32; 95% CI: 1.30-13.56). They have higher odds of undergoing uterine brace sutures and obstetric hysterectomy for the control of postpartum haemorrhage. Caesarean section at lower gestational age in women with placenta previa is associated with substantially greater blood loss (r=- 0.104). Foetal complications were overall high in the study, but remained comparable in both groups.

The preoperative haemoglobin concentration was comparable in both groups in our study (p=0.674). However, the blood loss was significantly higher in women with scarred uterus (p<0.05). Emergency caesarean section was associated with greater blood loss than planned caesarean section in women with an unscarred uterus; however, blood loss was similar in women with a scarred uterus. A substantially higher proportion of placenta accreta cases in women with a scarred uterus were managed as elective procedures, whereas fewer such cases occurred in emergency surgeries. As a result, the overall blood loss remained comparable between emergency and planned caesarean sections in women with placenta previa and a scarred uterus. Durukan et al., in their retrospective analysis, found that blood transfusion, maternal complication rates, and perinatal outcomes were significantly increased in women who were operated on emergency conditions due to placenta previa compared to planned cases [8]. However, they had a significantly higher number of placenta accreta spectrum cases operated in emergency than in planned caesarean section. Hence, scarring of the uterus or pathologic implantation of the placenta into the myometrium remains an independent risk factor for a greater amount of blood loss during caesarean section. Nevertheless, planned caesarean sections give us the opportunity to work out and arrange for multidisciplinary team care, including anaesthesia, transfusion medicine, urology, and intervention radiology in rare cases, optimise maternal preoperative condition, provide information and support to patients to help them make informed decisions, etc.

Zhang et al. discovered a non-linear relationship between prenatal haemoglobin levels and the overall blood transfusion rate in patients with placenta previa, emphasising the need to promote prenatal care for these patients, especially when there is a high likelihood of requiring blood transfusions [9]. Most of the patients who underwent emergency caesarean section were not booked at our institution and reported to an emergency triage area with antepartum haemorrhage, subsequently presenting with significant lower haemoglobin levels at admission (p=0.008) (data not shown here). Hence, the scope for the optimisation of haemoglobin preoperatively and multidisciplinary planning is compromised, leading to greater intraoperative and postoperative blood transfusion well corroborating the findings of Zhang et al. [9].

According to our study, women with scarred uterus and placenta previa had a higher odds of developing placenta accreta spectrum (OR=9.60; 95% CI: 1.14-20.39; p=0.014) compared to those with unscarred uterus. Placenta accreta spectrum refers to the abnormal invasion of the trophoblast, where part or all of the placenta penetrates the myometrium of the uterine wall [10]. The condition is primarily diagnosed through ultrasound, indicated by the disappearance of the normal clear zone interface between the uterus and the placenta, significant thinning of the underlying myometrium, and vascular changes in the placental lacunae and hypervascularity in the placental bed [6]. These ultrasound findings occur due to damage to the uterine wall, extending as far as the serosa, with placental tissue infiltrating the deep uterine circulation. Hence, such patients bleed profusely after the delivery of the baby. The overall incidence of placenta accreta spectrum in placenta previa in our study was 13.89%, with 3% occurring in women with unscarred uterus. A systematic review by Huang and Yang reported similar risk in women with no prior caesarean deliveries [11]. However, the incidence increases significantly with a history of one or more prior caesarean deliveries. The risk is reported to be 3%, 11%, 40%, 61%, and 67% for the first, second, third, fourth, and fifth or more caesarean sections, respectively [12]. The trophotropic theory suggests that the placenta migrates toward regions with a richer blood supply. Normally, it grows toward the fundus, which offers richer vascularisation. However, if the uterus has scarring or atrophy from previous surgeries or infections, blood supply to the endometrium can be disrupted, hindering differential growth in the lower uterine segment and limiting the upward migration of the placenta [2].

We observed a significantly higher BMI (p=0.021) in women with scarred uterus and hence its significant association with adverse pregnancy outcomes. Pregnancy obesity is linked to complications for both the foetus and the newborn. There is no single unifying mechanism responsible for the adverse outcomes related to maternal obesity. However, increased insulin resistance, inflammation, and oxidative stress associated with obesity have been attributed to early placental and foetal dysfunction [13]. There was a significant negative linear correlation (r=- 0.318; p=0.006) between gestational age and the amount of blood loss at delivery in our study. In an international cohort study by Butwick et al., it has been found that the risk of haemorrhage increases for women who deliver at a gestational age that deviates from the nadir, which is around 39 weeks [14]. Swedish women who delivered between 28 and 31 weeks had a higher adjusted odds of postpartum haemorrhage when compared to women delivering between 37 and 38 weeks [14]. Our discovery of significantly greater blood loss at lower gestational age in patients with defective placentation is supported by several studies [15,16]. The potential cause of such a finding could probably be due to premature vaginal bleeding necessitating emergency surgical interventions, inadequate formation of the lower uterine segment causing difficult delivery with subsequent uterine trauma, and abnormal placentation itself impeding the uterine contractility.

Women with unscarred uterus had higher intrauterine foetal deaths and poor neonatal Apgar scores at both first and fifth minutes of life in our study. A higher proportion of women in the unscarred group had emergency caesarean section in our study, and studies have revealed that neonatal outcomes are better in planned caesarean delivery in pregnancies complicated by abnormal placentation compared to emergency delivery. The reasons can be due to the mode of anaesthesia or the immediate predelivery maternal morbidities. General anaesthesia is more commonly used in emergency deliveries, and studies have shown general anaesthesia is linked to less favourable Apgar scores at one and five minutes after birth [8,11]. Secondly, the primary indication for emergency caesarean delivery in the studies reviewed is mostly repeated or excessive bleeding which can lead to severe maternal hypovolemic shock and a non-reassuring foetal status [17].

The strength of the study remains its prospective observational design which enabled systematic data collection, minimised recall bias, and ensured temporal association between uterine scarring and outcomes. Blood loss estimation using gravimetric methods, calibrated suction, and clot weighing enhanced accuracy compared with visual estimation. Inclusion of both emergency and planned caesarean sections reflects real-world practice and improves external validity. A broad range of clinically relevant maternal and neonatal outcomes was evaluated. However, the single-centre tertiary care setting limits generalisability to other healthcare levels. The limited study duration and use of convenience sampling may further affect external validity. Variations in surgeon expertise and intraoperative management could have influenced outcomes, introducing potential performance bias. Residual confounding from factors such as anaesthesia type, surgical techniques, and institutional protocols cannot be excluded. As a referral centre, the study population may have been enriched with complicated cases, potentially overestimating severe outcomes such as placenta accreta spectrum and massive haemorrhage.

## Conclusions

Hence, we conclude that scarred uterus with placenta previa can independently increase the risk of placenta accreta spectrum, postpartum haemorrhage, blood transfusion, and ICU admission. These women have higher odds of undergoing lifesaving interventions. Such interventions at a lower gestational age of delivery are associated with significant postpartum haemorrhage.
